# TTYH3 Modulates Bladder Cancer Proliferation and Metastasis via FGFR1/H-Ras/A-Raf/MEK/ERK Pathway

**DOI:** 10.3390/ijms231810496

**Published:** 2022-09-10

**Authors:** Polash Kumar Biswas, Yeonjoo Kwak, Aram Kim, Jaekwon Seok, Hee Jeong Kwak, Moonjung Lee, Ahmed Abdal Dayem, Kwonwoo Song, Jae-Yong Park, Kyoung Sik Park, Hyun Jin Shin, Ssang-Goo Cho

**Affiliations:** 1 Department of Stem Cell & Regenerative Biotechnology, Institute of Advanced Regenerative Science, Konkuk University, 120 Neungdong-ro, Gwangjin-gu, Seoul 05029, Korea; 2 Division of Biological Sciences, University of Montana, Missoula, MT 59812, USA; 3 Department of Urology, Konkuk University Medical Center, Konkuk University School of Medicine, Seoul 05029, Korea; 4 Department of Advanced Translational Medicine, Konkuk University, Seoul 05029, Korea; 5 School of Biosystems and Biomedical Sciences, College of Health Sciences, Korea University, Seoul 05029, Korea; 6 Department of Surgery, Konkuk University Medical Center, Konkuk University School of Medicine, Seoul 05029, Korea; 7 Department of Ophthalmology, Research Institute of Medical Science, Konkuk University Medical Center, Konkuk University School of Medicine, Seoul 05029, Korea

**Keywords:** bladder cancer, FGFR1, gene expression, MAPK, patient survival, TTYH3

## Abstract

Tweety family member 3 (TTYH3) is a calcium-activated chloride channel with a non-pore-forming structure that controls cell volume and signal transduction. We investigated the role of TTYH3 as a cancer-promoting factor in bladder cancer. The mRNA expression of *TTYH3* in bladder cancer patients was investigated using various bioinformatics databases. The results demonstrated that the increasingly greater expression of *TTYH3* increasingly worsened the prognosis of patients with bladder cancer. *TTYH3* knockdown bladder cancer cell lines were constructed by their various cancer properties measured. *TTYH3* knockdown significantly reduced cell proliferation and sphere formation. Cell migration and invasion were also significantly reduced in knockdown bladder cancer cells, compared to normal bladder cancer cells. The knockdown of *TTYH3* led to the downregulation of H-Ras/A-Raf/MEK/ERK signaling by inhibiting fibroblast growth factor receptor 1 (FGFR1) phosphorylation. This signaling pathway also attenuated the expression of c-Jun and c-Fos. The findings implicate TTYH3 as a potential factor regulating the properties of bladder cancer and as a therapeutic target.

## 1. Introduction

Bladder cancer is the 10th most common cancer worldwide, accounting for 3.0% of all cancer diagnoses and 2.1% of cancer-related deaths [1]. Bladder cancer is classified into non-muscle-invasive bladder cancer (NMIBCa) and muscle-invasive bladder cancer (MIBCa). Seventy-five percent of bladder cancer patients are diagnosed with NMIBCa, where the tumor remains in the mucosa. The remaining 25% are diagnosed with MIBCa, where the tumor has penetrated the bladder muscle and spread to lymph nodes or surrounding organs, with a poor prognosis [2,3,4]. The most commonly used treatment modality for patients with NMIBCa is transurethral resection of bladder tumors (TURBT). TURBT is sometimes combined with intravesical instillation to prevent recurrence and progression. In the case of MIBCa, penetration of the tumor into bladder muscle often necessitates radical removal of the bladder (RC). Although many surgical improvements and advances in chemotherapy combined with surgery have been made, the survival rates for bladder cancer have remained unchanged for decades [5]. Therefore, a variety of advanced molecular studies, such as identification of therapeutic gene targets and individualized therapies, are important to complement current surgical therapies.

The relationship between cancer and ion channels has been studied in several ways. Among the various tissues, the bladder is associated to various nerves and is involved in urine storage and voiding through “afferent function” and “efferent function”; therefore, the role of ion channels is important. The urothelium works with the suburothelium to contract and relax [6]. Urinary bladder smooth muscle (UBSM) relaxation and contraction depend on UBSM excitability, which is regulated by various ion channels, including Ca^2+^, K^+^, and Cl^−^ channels [7]. Mammalian cells express a variety of ion channels on the membrane surface that can selectively transport specific ions and control various functions in the host. For example, Na^+^, K^+^, and Ca^2+^ channels control the electrical response of cells, and Cl^−^ channels control the membrane potential and cell volume [8]. These activities are regulated by various physical and chemical processes. Ion channels can even modulate important signaling mechanisms of cancer progression, such as cell proliferation, migration, and apoptosis [9]. Recent evidence demonstrated that certain ion channel inhibitors can induce growth arrest in cancer cells [10]. The investigation of these channels is a promising avenue of research into molecular diagnoses. Furthermore, there is a close interaction between Ca^2+^ and Cl^−^ flux, which may affect brain tumor development. Cl^−^ intracellular channels (CLIC) are highly expressed in gliomas and are associated with enhanced cell proliferation and migration [11]. Calcium-activated chloride channels, such as that formed by the Anoctamin 1 (ANO1) protein, frequently amplified and played an important role in various cancers, such as breast cancer, ovarian cancer, and bladder cancer [12,13,14,15].

*Tweety* family member 3 *(TTYH3)* encodes the TTYH3 protein that forms calcium-activated chloride channels. *TTYH3* is highly expressed in excitatory tissues [16]. *TTYH3* encodes the third member of *tweety* mammalian homolog. TTYH3 is a large-conductance Ca2^+^-activated chloride channel with a non-pore-forming structure [17,18,19]. We previously reported that *TTYH3* is highly expressed in gastric cancer [20]. We compared *TTYH3* expression in normal and gastric cancer tissues using various databases. *TTYH3* expression was higher in gastric cancer tissues than in normal tissues. A high expression of *TTYH3* led to poor outcomes in patients with gastric cancer. Additionally, a recent study has shown that *TTYH3* overexpression promotes cell proliferation, migration, and invasion and inhibits apoptosis in hepatocellular carcinoma [21]. Previous studies have shown that *TTYH3* is overexpressed in certain cancers and is related to a poor prognosis of cancer [20,21]; however, its biological function and clinical significance in bladder cancer has never been studied.

Here, we investigated the correlation between TTYH3 and bladder cancer and revealed the mechanism of its effect on bladder cancer through experimental analyses. The expression of *TTYH3* and its prognosis in bladder cancer were assessed in vitro and clinically using knockdown cells and bioinformatics web tools, respectively. The functions of TTYH3 in bladder cancer cell proliferation, sphere formation, migration, and invasion were also assessed in vitro. Finally, we investigated the molecular mechanisms that influence bladder cancer prognosis.

## 2. Results

### 2.1. Expression of TTYH3 Is Associated with Decreased Survival Rates in Bladder Cancer Patients

**Figure 1:**
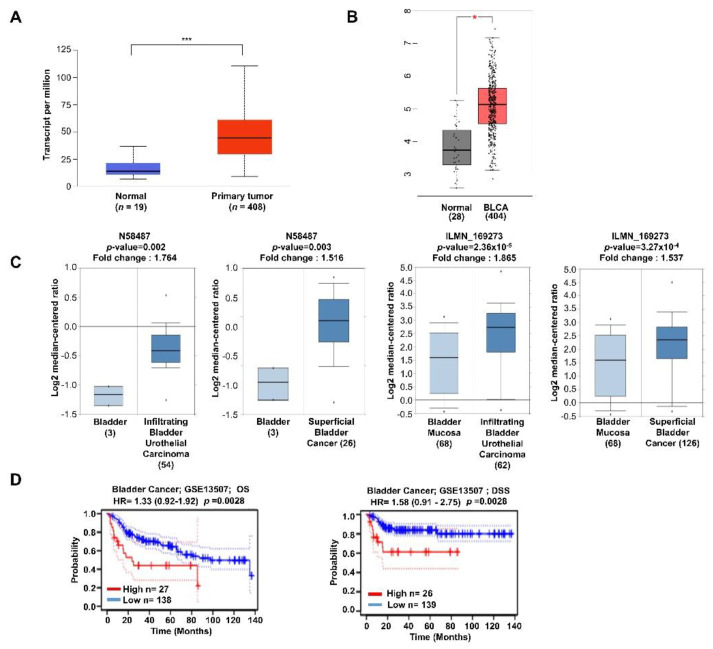
Analysis of *TTYH3* mRNA expression pattern and prognosis in bladder cancer. (**A**) Comparison of the expression of *TTYH3* in normal and bladder urothelial carcinoma (BLCA) tissues using the UALCAN database. Red boxes represent tumor tissues and blue boxes represent normal tissues. Red and blue dashed lines represent the average expression value of all tumor and normal tissues, respectively. (**B**) Comparison of the expression of *TTYH3* in normal and BLCA tissues using the GEPIA2 database. Red boxes represent tumor tissues and gray boxes represent normal tissues. (**C**) Analysis of *TTYH3* expression levels in bladder cancer versus normal tissues on the Oncomine dataset. The threshold was designed using the following specific parameters: *p*–value = 1 × 10^5^ and fold change = 1.5. (**D**) The graph compares the survival rate of high (red) and low (blue) expression of TTYH3 in bladder cancer patients using the PrognoScan database. (* *p* ≤ 0.05, *** *p* ≤ 0.001).

**Figure A1:**
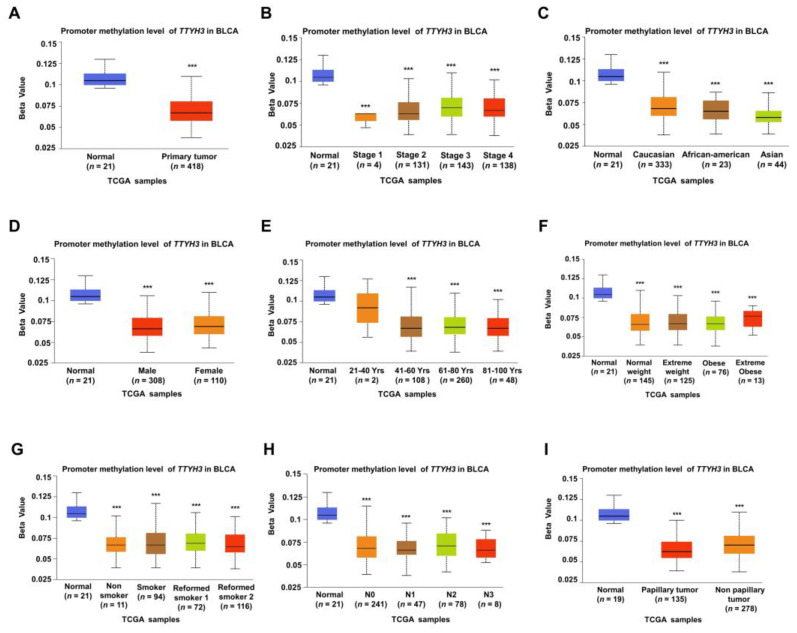
Promoter methylation of TTYH3 gene is significantly down-regulated in bladder cancer tissue. (**A**) The Cancer Genome Atlas (TCGA) clinical data from the UALCAN web tool were used to compare promoter methylation of TTYH3 in normal and bladder cancer patient tissues. The data were categorized based on individual cancer stages (**B**), patient’s race (**C**), patient’s gender (**D**), patient’s age (**E**), patient’s weight (**F**), smoking status (**G**), nodal metastasis status (**H**), histological subtypes (**I**). (*** *p* ≤ 0.001).

The bladder has many nerves and ion channels that control urination, with various ions and ion channels having important roles in the bladder. The link between ion channels and cancer cell progression is attracting attention [11,14]. Therefore, we investigated the relationship between *TTYH3* expression and prognosis in bladder cancer patients. Various web-based tools, such as UALCAN, GEPIA2, Oncomine, and PrognoScan, were used to determine whether *TTYH3* gene expression affects cancer progression in bladder cancer patients. We compared the mRNA expression of bladder urothelial carcinoma (BLCA) samples with their normal counterparts in The Cancer Genome Atlas (TCGA) dataset, using the UALCAN publicly available cancer omics database tool (Figure 1A). We also compared mRNA expression levels using the GEPIA2 web tool (Figure 1B). In both datasets from different web tools, mRNA expression of *TTYH3* was higher in bladder cancer tissues than in normal tissues. Using the Oncomine cancer microarray database, the mRNA expression of *TTYH3* in two types of bladder cancer, infiltrating BLCA and superficial bladder cancer, was compared with that in a healthy bladder. The Blaveri Bladder 2 and Lee Bladder datasets were used (Figure 1C). PrognoScan was used for meta-analysis of the prognostic values of genes. The survival rates according to the increase in *TTYH3* gene expression in patients with bladder cancer were compared. Increasingly greater expression of the *TTYH3* gene was associated with increasingly worse prognosis of bladder cancer patients (Figure 1D). A positive correlation was evident between high expression of *TTYH3* and poor outcomes. To evaluate the methylation level of the *TTYH3* promoter in bladder cancer, the TCGA dataset was analyzed using the UALCAN web tool. The analysis revealed downregulation of promoter methylation of *TTYH3* in BLCA, irrespective of clinicopathological characteristics, such as individual cancer stage, race, sex, age, weight, smoking status, nodal metastasis status, and histological subtype (Figure A1).

### 2.2. TTYH3 Knockdown Suppresses Tumor Growth and Proliferation in Bladder Cancer Cells

**Figure 2:**
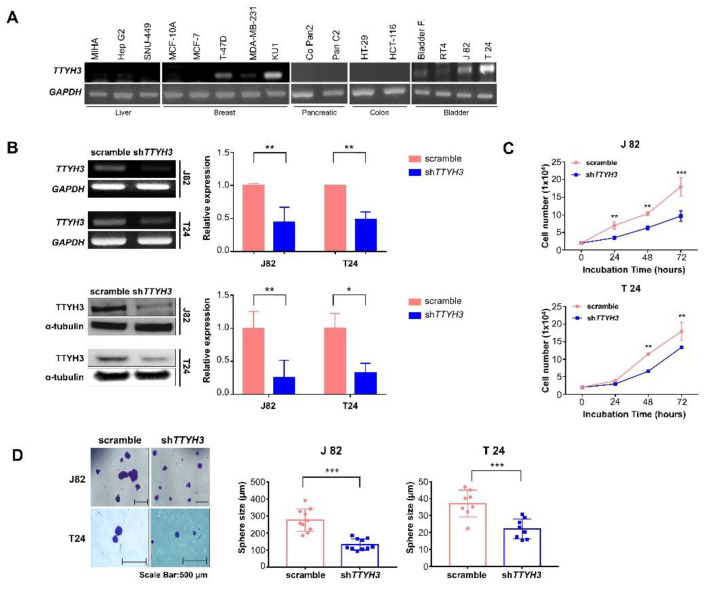
Knockdown of *TTYH3* inhibits proliferation and sphere formation of bladder cancer cells. (**A**) Expression of *TTYH3* in various cancer cell lines and normal cell lines. Normal liver cell line (MIHA); liver cancer cell lines (Hep G2 and SNU-449); normal breast epithelial cell line (MCF 10A); breast cancer cell lines (MCF7, T-47D, MDA-MB-231, and KU1 (breast cancer stem-like cells/tumor-initiating cells)); pancreatic cancer cell lines (Capan-2 and Panc2); colon cancer cell lines (HT-29 and HCT 116); normal bladder cell line (primary bladder fibroblast cells); bladder cancer cell lines (RT4, J82, and T24). Glyceraldehyde 3-phosphate dehydrogenase (GAPDH) was used as the loading control. (**B**) *TTYH3* expression in scramble and short hairpin (sh)*TTYH3*-transduced cells was analyzed by RT-PCR and western blot analysis in J82 and T24 bladder cancer cell lines. *GAPDH* or α-tubulin expression was used as the control. The band density was measured using ImageJ software and the values were presented in graph. (**C**) Effect of *TTYH3* knockdown on cell proliferation was analyzed by counting of each group of cells for 3 days. (**D**) Sphere-forming ability of scramble and sh*TTYH3* transduced cells. The size of the sphere was measured using ImageJ software and presented in graph. The scale bar denotes 500 µm. All statistical analyses were performed with two-way ANOVA; * *p* ≤ 0.05, ** *p* ≤ 0.01, and *** *p* ≤ 0.001.

To confirm that *TTYH3* expression affects bladder cancer function, we performed some in vitro assays in bladder cancer cell lines. The expression of *TTYH3* was compared in various cancer cell lines. The expression of *TTYH3* was higher in bladder cancer cell lines than in other cancer cell lines and higher in cell lines derived from high-grade transitional cell carcinomas, such as T24 and J82 cell lines (Figure 2A). We were interested in investigating the mechanisms involved in *TTYH3* expression in bladder cell lines. To understand the role of TTYH3 in bladder cancer cells, we used a lentiviral *TTYH3* knockdown vector to suppress *TTYH3* expression in J82 and T24 cells. The knockdown of *TTYH3* was confirmed by reverse transcription-polymerase chain reaction (RT-PCR) (Table 1). *TTYH3* expression was reduced by more than half in J82 and T24 cells. TTYH3 protein expression was assessed by western blotting. The protein expression was also reduced by approximately 50% in J82 and T24 cells (Figure 2B). Cancer cell growth between control and *TTYH3* knockdown cells for 3 days was compared using a cell counting assay. The number of surviving cells was significantly reduced by *TTYH3* knockdown on days 1, 2, and 3 in the J82 cell line and significantly decreased on days 2 and 3 in the T24 cell line (Figure 2C). In addition, the sphere-forming assay showed that *TTYH3* knockdown J82 and T24 cells formed smaller spheres than control cells (Figure 2D). 

### 2.3. TTYH3 Involvement in a Variety of Signaling Pathways That Regulate Cellular Functions, including MAPK Signaling

**Figure 3:**
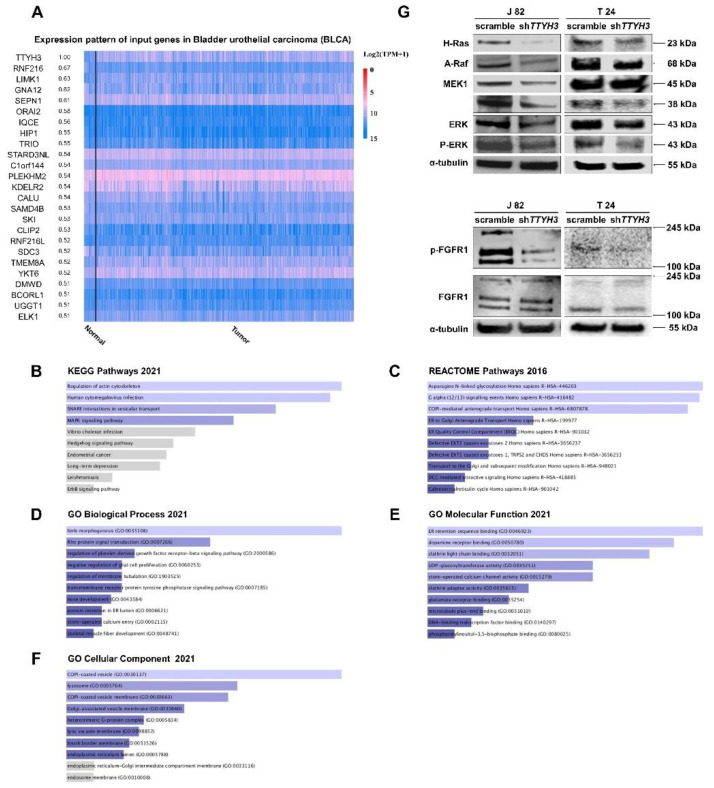
Genes co-expressed with *TTYH3* and related pathways in bladder cancer. (**A**) UALCAN analysis of the profile of genes co-expressed with *TTYH3*. (**B**–**F**) Profiles of genes co-expressed with *TTYH3* involved in signaling pathways in bladder urothelial carcinoma (BLCA). This figure depicts the results showing the gene ontology (GO) and signaling pathways of *TTYH3*, and the positively correlated genes in bladder cancer. The bar graphs represent genes positively correlated to *TTYH3*, showing the involvement in BLCA in pathway analysis performed using Enricher. The bar graph represents the ratio of the percent composition of terms in proteomic data vs. percent composition in the genome annotation. The length of the bar represents the significance of that specific gene-set or term. (**G**) Western blot analysis of mitogen-activated protein kinase (MAPK) signaling and FGFR1 proteins in *TTYH3*-knockdown bladder cancer cell lines. α-tubulin expression was used as the control.

To identify the molecular mechanisms affected by *TTYH3* expression, genes positively correlated with *TTYH3* expression were identified using the UALCAN data tool. The 24 most relevant genes were selected (Figure 3A). Pathway and ontology analyses involving *TTYH3* and the 24 positively associated genes were performed using Enrichr (https://maayanlab.cloud/Enrichr (accessed on 19 December 2021)). Kyoto Encyclopedia of Genes and Genomes (KEGG) pathway analysis revealed regulation of the actin cytoskeleton, cytomegalovirus infection, SNARE interaction, and mitogen-activated protein kinase (MAPK) signaling pathway functions when *TTYH3* co-expressed genes were highly expressed (Figure 3B). REACTOME pathway analysis strongly correlated glycosylation, G-alpha signaling, and transport-related signaling with *TTYH3* co-expressed genes (Figure 3C). Gene ontology (GO) analysis revealed that genes involved with binding functions or constituents of vesicles and membranes accounted for a high proportion of genes related to *TTYH3* (Figure 3D–F). Since TTYH3 is a transmembrane channel, vesicle transport system pathways are associated with the expression of *TTYH3*. In addition, KEGG analysis confirmed the relationship of the expression of *TTYH3* and the MAPK signaling pathway (Figure 3B). In *TTYH3* knockdown cell lines, the protein levels of H-Ras, A-Raf, phospho-mitogen-activated protein kinase kinase (MEK), and phospho-extracellular signal-regulated kinase (ERK) decreased in both T24 and J82 cell lines. In addition, levels of phosphorylated fibroblast growth factor receptor 1 (FGFR1) were decreased in the knockdown cell lines (Figure 3G). The findings indicate that the expression of *TTYH3* may affect the phosphorylation of FGFR1, inhibit H-Ras/A-Raf/MEK signaling, and inhibit the proliferation of bladder cancer cells.

### 2.4. TTYH3 Knockdown Inhibits Bladder Cancer Cell Migration and Invasion

**Figure 4:**
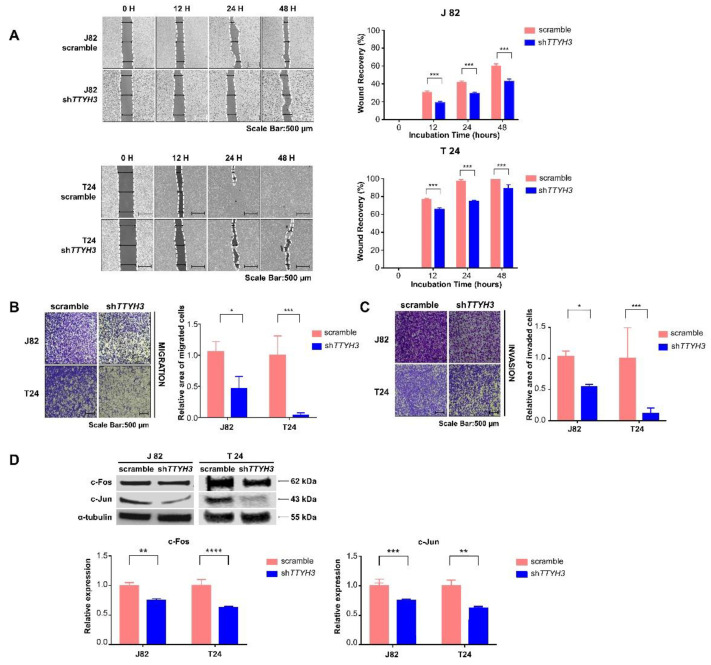
Decreased migration and invasion of *TTYH3* knockdown bladder cancer cells. (**A**) Cell mobility measured by a wound healing assay. The uncovered areas in the wound healing assays were quantified as a percentage of the original wound area. The area was measured using T-Scratch. Scale bar denotes 500 µm. (**B**,**C**) The effect of *TTYH3* knockdown on cell migration and invasion determined by transwell migration assay and basement membrane extract cell invasion assay. Quantifications of migrating or invading cells of each cell line are shown as proportions of their vector controls. Scale bar denotes 500 µm. (**D**) Western blot analysis of c-Fos and c-Jun proteins in TTYH3-knockdown bladder cancer cell lines. α-tubulin expression was used as the control. Bar graphs show the statistical analysis of three independent experiments. All the statistical analyses were performed with two-way ANOVA; * *p* ≤ 0.05, ** *p* ≤ 0.01, *** *p* ≤ 0.001, and **** *p* ≤ 0.0001.

We also confirmed the progression of bladder cancer using migration and invasion assays and bladder cancer cells. By observing the behavior of cancer cells in vitro, the migration and invasion ability of *TTYH3* knockdown bladder cancer cells were compared with those of scramble-transfected bladder cancer cells. The collective and individual migration abilities of bladder cancer cells, in which *TTYH3* was knocked down, were also determined using wound closure, and transwell migration assays were performed (Figure 4A,B). When wound closure rates were measured at 12, 48, and 72 h, migration was significantly decreased when *TTYH3* was knocked down in both T24 and J82 cell lines (Figure 4A). After seeding the cells in the apical chamber of the transwell, the cells were incubated for 24 h. Cells that passed through the pores of the chamber were stained and measured. The migration ability of the bladder cancer cells was reduced upon *TTYH3* knockdown (Figure 4B). The invasion ability of bladder cancer cells was reduced in *TTYH3* knockdown cells in a basement membrane extract cell invasion assay. Invasion assays were performed using transwell apical chambers coated with Matrigel as an extracellular matrix to evaluate the early metastatic ability of the cancer cells. The proportion of cells that passed through the membrane and invaded the basal chamber was decreased in knockdown cells (Figure 4C). Transcription factors, c-Jun and c-Fos, regulate cell migration and invasion by binding to the AP-1 binding site [22,23]. Both c-Fos and c-Jun are upregulated in bladder cancer and have a critical role in tumor progression [24]. In the present study, when *TTYH3* was knocked down, the expressions of c-Fos and c-Jun decreased, and the abilities to migrate and invade bladder cancer were reduced (Figure 4D).

## 3. Discussion

Predicting tumor recurrence and metastasis is essential to improve the prognosis of patients with bladder cancer. In addition, the detection of novel molecular biomarkers is essential for better bladder cancer treatment. Recent data have shown that changes in cell volume with chloride accumulation affect tumor proliferation and migration [25]. Ion channels are functionally important in smooth muscle tissues, including the bladder [9]. The *tweety* family of genes (*TTYH*s) are chloride channel-responsive genes that contribute to several cellular processes, including cell adhesion, cell division, tumorigenesis, and regulation of calcium activity. TTYH3 is also a large-conductance Ca^2+^-activated chloride channel whose expression results in poor prognosis in gastric cancer [20].

We observed that *TTYH3* was significantly upregulated in bladder cancer tissue, compared to adjacent normal tissue. In patients with BLCA, the elevated *TTYH3* levels correlated with poor prognosis in bladder cancer patients. These findings indicate that TTYH3 may promote the growth and progression of bladder cancer. Based on the upregulation of *TTYH3* in BLCA in our study, we conducted molecular experiments and explored the potential biological role of TTYH3 in vitro. We knocked down *TTYH3* using lentivirus in the J82 and T24 bladder cancer cell lines. *TTYH3* knockdown significantly inhibited the growth, migration, and invasion of bladder cancer cells. Cell proliferation and sphere-forming ability were reduced when *TTYH3* was knocked down. Moreover, migration and invasion abilities were reduced in *TTYH3* knockdown bladder cancer cell lines.

**Figure 5:**
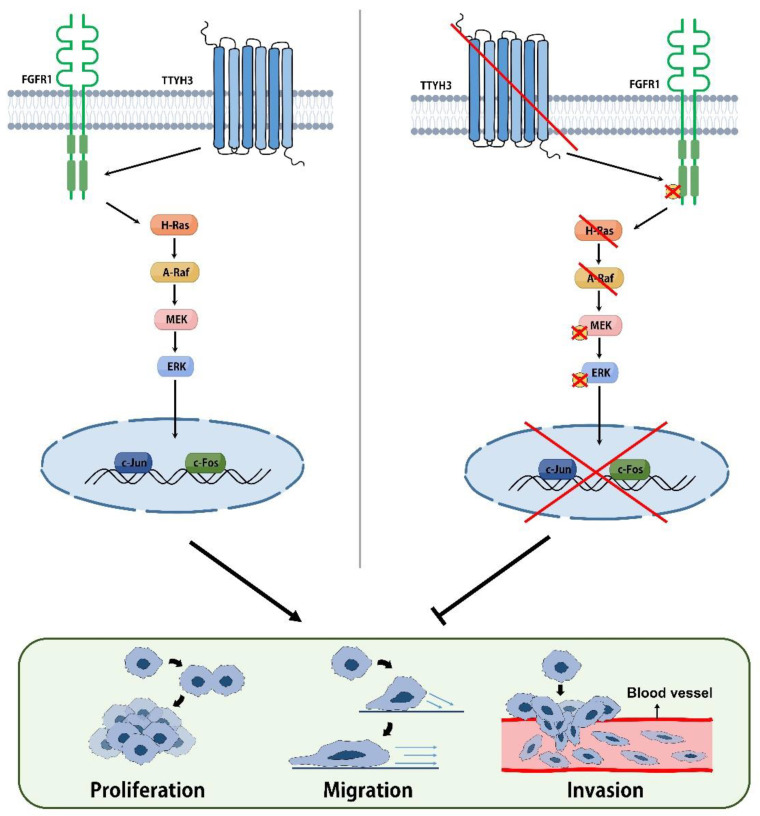
Schematic diagram of the proposed role of TTYH3 in bladder cancer. *TTYH3* knockdown regulates cell proliferation, migration, and invasion by inhibiting FGFR1 and MAPK signaling.

Using the UALCAN web tool, we also found a downregulation of the promoter methylation of *TTYH3* in bladder cancer tissues. Because of the low promoter methylation of *TTYH3*, its expression of *TTYH3* in bladder cancer tissues is predicted to be higher than that in normal tissues. Further studies are needed to determine whether *TTYH3* promoter methylation affects *TTYH3* expression and prognosis in bladder cancer. Bioinformatics analysis of the UALCAN dataset showed that genes correlated with *TTYH3* in BLCA were significantly associated with the MAPK signaling pathway. Furthermore, TTYH3 affected the activity of MAPK signaling, since TTYH3 inhibition markedly impaired the MAPK pathway. The tumorigenic properties of TTYH3 shown in our results proceed through the Ras/Raf pathway. The proliferation, migration, and invasion of bladder cancer cells are regulated by FGFR1 and MAPK signaling. We investigated the Ras/Raf/MEK/ERK pathway, which is associated with cancer cell proliferation [26]. H-Ras mutations are common in bladder cancer, and H-Ras modulates ERK-mediated pathways to regulate bladder cancer proliferation and survival [27,28]. We observed changes in the ERK-mediated H-Ras pathway in *TTYH3* knockdown bladder cancer cells. In these cells, the protein levels of H-Ras, A-Raf, phospho-MEK1/2, and phospho-ERK decreased in both T24 and J82 cell lines. The expression of FGFR1, which promotes the ability of cancer cells through the Ras-ERK pathway [29], was also confirmed. The collective findings indicate that bladder cancer cells are regulated by TTYH3 through FGFR1 and the H-Ras/A-Raf/MEK/ERK pathways (Figure 5).

Recent studies have shown that this signaling is affected by growth factor receptors, such as EGFR and FGFR [30]. FGFR1 is a major member of the FGFR family and is crucial in a variety of solid tumors. Specific ligands that bind FGFR1 induce various downstream signaling cascades, including RAS, MEK, and ERK. FGFR1 regulates cell proliferation, differentiation, migration, and angiogenesis through this signaling [31]. TTYH3 affected H-Ras/A-Raf/MEK signaling by regulating the phosphorylation of FGFR1. However, the direct relationship between TTYH3 and the FGFR1 signaling pathway requires further research.

Our study has some limitations. First, although many types of MAPK signaling regulate cancer properties, this study focused on the mechanism of the H-Ras/MEK/ERK pathway and FGFR1 phosphorylation in bladder cancer. A recent study indicated that calcium-activated chloride channel upregulation is associated with the ligand-dependent EGFR signaling pathway [14]. Second, although we used bioinformatics analysis to determine *TTYH3* promoter methylation level, for further study, we need to analyze the association between *TTYH3* promoter methylation level and *TTYH3* expression level to confirm if they are significantly correlated in clinical samples. Third, in vivo experiments are required for in-depth verification. We investigated the mechanism of TTYH3 in bladder cancer through in vitro experiments and analysis of clinical data using bioinformatics tools. In vivo experiments in animal models were not done. Further animal model investigations are required to verify and improve our findings.

Our study indicates that TTYH3 regulates tumor proliferation, migration, and invasion and is overexpressed in bladder cancer patients, providing poor outcomes. Furthermore, we present the possibility that TTYH3 can act as a predictive biomarker in bladder cancer patients and can be used as a therapeutic target. Consequently, our data revealed that TTYH3 might function as a favorable tumor activator gene for bladder cancer and that the molecular basis underlying the TTYH3 and FGER1 mediated the MAPK signaling, which can be utilized in MAPK-based bladder cancer therapeutics approach and might hold potential clinical targets.

## 4. Materials and Methods

### 4.1. Cell Culture

J82 and T24 human bladder cancer cell lines and HEK 293T embryonic kidney cells were obtained from American Type Culture Collection (ATCC, Manassas, VA, USA). The cells were cultured in RPMI-1640 medium and high-glucose Dulbecco’s modified Eagle medium (DMEM) supplemented with 10% fetal bovine serum (FBS) (Gibco™, Thermo Fisher Scientific, Waltham, MA, USA) and 1% penicillin-streptomycin (Gibco™, Thermo Fisher Scientific), respectively, in a humidified incubator with an atmosphere of 5% CO_2_ at 37 °C.

### 4.2. TTYH3 Knockdown Using Lentiviral Vector

To produce lentivirus, HEK 293T cells were transfected using lentiviral packaging (psPAX2), envelop (pCMV-VSV-G) plasmids, and PolyJet in vitro DNA transfection reagent (Signogen, Burkina Faso, Africa), following the manufacturer’s recommended protocol. The shRNA targeting sequencing for *TTYH3* was: 5′-CATGAGCCAGAACGCTAATTT-3′ (sh*TTYH3*). The vector was transfected in serum-free medium for 3 h and then incubated in a culture medium containing 5% FBS. After 12 h, the medium was replaced with a culture medium containing 10% FBS. The supernatants were collected 48–72 h after transfection and filtered using a 0.45 µm syringe filter to collect the lentiviral sup. The J82 and T24 bladder cancer cell lines were infected with lentiviral sup diluted 3-fold in culture medium, supplemented with 8 µg/mL hexadimethrine bromide in 60 mm dishes. In the next passage, selection was performed in medium supplemented with 6 µg/mL of puromycin.

### 4.3. RNA Isolation and RT-PCR Analysis

Total RNA was extracted using a Labozol Reagent RNA Extraction kit (Cosmogenetech, Seoul, Korea). The purified total RNA (2 µg) was reverse transcribed using a cDNA synthesis kit (Promega, Madison, WI, USA) according to the manufacturer’s instructions. RT-PCR was conducted using r-Taq plus Master Mix (Elpis Biotech, Seoul, Korea). The PCR products were separated by agarose gel electrophoresis and quantified using Image J software (https://imagej.net (accessed on 29 May 2021)).

### 4.4. Western Blotting

For protein extraction, cells were lysed with RIPA buffer (CBR002; LPS Solution, Daejeon, South Korea) with Halt™ Protease Inhibitor Cocktail (100×, 87,786; Thermo Fisher Scientific). Protein samples were loaded onto 10% SDS-PAGE gels and analyzed using the iBlot™ 2 Dry Blotting System (Invitrogen, Carlsbad, CA, USA). Membranes were incubated with primary antibodies against TTYH3 (ARP49946_P050, 1:1000 dilution; Aviva Systems Biology Corporation, San Diego, CA, USA), H-Ras (sc-35, 1:1000 dilution; Santa Cruz Biotechnology, Dallas, TX, USA), A-Raf (4432, 1:1000 dilution; Cell Signaling Technology, Beverly, MA, USA), MEK1 (sc-6250, 1:1000 dilution; Santa Cruz Biotechnology), p-MEK1 (sc-7995, 1:1000 dilution; Santa Cruz Biotechnology), ERK1/2 (05-1152, 1:1000 dilution; Cite Ab, Bath, England), p-ERK (sc-7383, 1:1000 dilution; Santa Cruz Biotechnology), FGFR1 (NBP2-33784, 1:500 dilution; Novus Biologicals, MN, USA), and p-FGFR1 (44–1140 G, 1:500 dilution; Invitrogen) at 4 °C overnight. Each membrane was then washed twice with Tris-buffered saline-Tween buffer at 10 min intervals. Secondary antibodies included anti-mouse (sc-2005, 1:3000 dilution; Santa Cruz Biotechnology), goat IgG (sc-2020, 1:3000 dilution; Santa Cruz Biotechnology), and rabbit IgG (sc-2004, 1:3000 dilution; Santa Cruz Biotechnology). These antibodies were tagged with horseradish peroxidase (HRP). The antibodies were bound to the membrane at room temperature for 2 h. After washing in the same manner as the primary antibody, the specific proteins in the membranes were analyzed using an ECL detection kit (Amersham Bioscience, Piscataway, NJ, USA). Quantification of protein bands was analyzed using an Invitrogen™ iBright™ imaging system (iBright CL1000, Thermo Fisher Scientific).

### 4.5. Cell Proliferation

J82 and T24 cells were seeded in 12-well plates at 2 × 10^4^ cells/well and incubated at 37 °C in 5% CO_2_ for 24, 48, and 72 h. Cell counting was performed using a hemocytometer following staining with Trypan blue (Gibco™ 15250061, Thermo Fisher Scientific).

### 4.6. Sphere-Forming Assay

Scramble and sh*TTYH3* T24 and J82 cells were seeded at density of 5 × 10^4^–1 × 10^5^ in six uncoated wells filled with DMEM/F12 supplemented with B27-supplement (1:50; Invitrogen) and also supplemented with 20 ng/mL epidermal growth factor (Sigma-Aldrich, St. Louis, MO, USA), 10 μg/mL insulin (Invitrogen), and 0.4% bovine serum albumin (Sigma-Aldrich). The cells were incubated at 37 °C in 5% CO_2_ for 5 days then collected in a conical tube. After incubation at room temperature for 5 min, cells were gently spun down and the supernatant removed. Then, after staining with 0.125% crystal violet for 3–5 min, dilute with PBS. The stained spheres were transferred to a plate and observed.

### 4.7. Wound Closure Assay

For the wound healing/migration test, 1 × 10^6^ cells were seeded in a 60 mm cell culture plate and grown until 90% confluence. The cells were then incubated with mitomycin C (10 µg/mL) to inhibit cell division. Cells were cultured in 5% CO_2_ at 37 °C. After the incubation period, the monolayer of cells was wounded with a 200 μL pipette tip. The wound areas were measured every 24 h and the filled area by moved cells was evaluated using TScratch software. Wound recovery levels were quantified as follows: recovered area at 12, 24, 48 h each divided by initial wound area at 0 h. Then, the values were plotted as percentages and graphed.

### 4.8. Transwell Assay

Cell migration and invasion capacities were measured using transwell chambers (Corning Life Sciences, New York City, NY, USA). For the migration assay, 3.5 × 10^5^ bladder cancer cells were seeded into a transwell apparatus (6.5 mm) equipped with sterile 8.0 um pore polycarbonate membrane inserts. An amount of 100 µL serum-free medium was added in 12-well insert, while 600 µL medium with 1% FBS was placed into the lower chamber. For invasion assay, the 12-well insert was coated with matrigel for 24 h and 1 × 10^5^ bladder cancer cells were seeded. After incubation for 24 h, the transwell membrane was fixed with 4% paraformaldehyde solution for 20 min followed by 100% methanol treatment for 20 min. After fixation, 0.5% crystal violet staining was conducted for 20 min. The area of the migrated and invaded cells was measured using ImageJ software and graphed.

### 4.9. Statistical Analysis

All experiments were conducted at least three times. The data are presented as mean ± standard deviation. For statistical analysis, an unpaired t-test was performed between two groups (control vs. treated). Statistical significance was judged according to the experiment (* *p* ≤ 0.05, ** *p* ≤ 0.01, *** *p* ≤ 0.001, *** *p* ≤ 0.0001).

### 4.10. Bioinformatics Database Analysis

UALCAN (http://ualcan.path.uab.edu (accessed on 18 December 2021)), GEPIA2 (http://gepia2.cancer-pku.cn (accessed on 19 December 2021)), Oncomine (https://www.oncomine.org (accessed on 19 December 2021)), and PrognoScan (http://dna00.bio.kyutech.ac.jp/PrognoScan/ (accessed on 19 December 2021)) were used in this study.

#### 4.10.1. UALCAN Database Analysis

UALCAN is an interactive data web-portal to analyses of The Cancer Genome Atlas (TCGA) gene expression data. It provides analysis of the relative expression between tumor and normal samples in various tumor subgroups [32,33]. We used UALCAN to understand genes, proteins, and related pathways in cancer. We used the UALCAN data web to determine the expression level of the TTYH3 gene and the level of promoter methylation in bladder cancer. Moreover, UALCAN was used to search for genes that are upregulated together when TTYH3 is upregulated and to search for gene-related functions and pathways in cells.

#### 4.10.2. GEPIA2 Database Analysis

GEPIA2 is an updated version of GEPIA (Gene Expression Profiling Interactive Analysis) web server, based on TCGA and the GTEx databases. GEPIA2 was used to compare mRNA expression of TTYH3 in GTEx bladder cancer samples and TCGA normal samples with fold change of log2 (FC) cutoff 1 and *p*-value < 0.01.

#### 4.10.3. Oncomine Database Analysis

Oncomine is a web-based database, including public cancer microarray data [34]. The mRNA expression pattern of *TTYH3* was analyzed in bladder cancer using Oncomine. *TTYH3* expression between normal bladder tissue and bladder urothelial cancer tissue was compared. Analysis was based on a *p*-value < 0.005 and a fold change of 1.5 times or more.

#### 4.10.4. PrognoScan Database Analysis

PrognoScan, a database for meta-analysis of prognostic values of genes, provides cancer microarray datasets with clinical data. The survival rate of bladder cancer patients according to the expression level of the *TTYH3* gene was analyzed using PrognoScan, and data with a *p*-value < 0.005 were included.

## 5. Conclusions

In conclusion, *TTYH3* expression is increased in bladder cancer and is related to the aggressive progression of cancer. *TTYH3* affects the phosphorylation of FGFR1 and the reduction of H-Ras/A-Raf/MEK signaling. The knockdown of *TTYH3* inhibits bladder cancer progression.

## Figures and Tables

**Table 1:** RT-PCR primers.

Species	Target	Forward Primer	Reverse Primer
Human	*TTYH3*	5′-GACGCCTACGTGACCAAAAT-3′	5′-GACGGTCCTCAGAAGCTCAG-3′
	*GAPDH*	5′-AATCCCATCACCATCTTCCAG-3′	5′-CACGATACCAAAGTTGTCATG-3′

## Data Availability

Not applicable.
